# Supplementary material for: Discovery and application of insertion-deletion (INDEL) polymorphisms for QTL mapping of early life-history traits in Atlantic salmon
Source: BMC Genomics. 2010 Mar 8;11:156. doi: 10.1186/1471-2164-11-156 (PMC2838853; doi:10.1186/1471-2164-11-156)
Supplement: Additional file 2 — Information on developed 76 locus single-run INDEL panel in Atlantic salmon. Information on fluorescence labeling, primer concentrations, PCR pooling and links to alignments, INDEL motifs and GENESCAN (Burge and Karlin 1997) predictions of genes/exons are available in html format. [file 1471-2164-11-156-S2.ZIP › Additionalfile2/table7583.html]

Contig Alignment

```
Base	A B C D E F G H I J K   
1	G . . . . . . . . . .    
2	G . . . . . . . . . .    
3	G . . . . . . . . . .    
4	G . . . . . . . . . .    
5	C . . . . . . . . . .    
6	A . . . . . . . . . .    
7	A . . . . . . . . . .    
8	T . . . . . . . . . .    
9	A . . . . . . . . . .    
10	T . . . . . . . . . .    
11	G . . . . . . . . . .    
12	G . . . . . . . . . .    
13	T . . . . . . . . . .    
14	T . . . . . . . . . .    
15	G . . . . . . . . . .    
16	C . . . . . . . . . .    
17	G . . . . . . . . . .    
18	T . . . . . . . . . .    
19	A . . . . . . . . . .    
20	G . . . . . . . . . .    
21	A . . . . . . . . . .    
22	T . . . . . . . . . .    
23	C . . . . . . . . . .    
24	T . . . . . . . . . .    
25	G . . . . . . . . . .    
26	T . . . . . . . . . .    
27	C . . . . . . . . . .    
28	C . . . . . . . . . .    
29	A . . . . . . . . . .    
30	G . . . . . . . . . .    
31	C . . . . . . . . . .    
32	T . . . . . . . . . .    
33	A . . . . . . . . . .    
34	G . . . . . . . . . .    
35	T . . . . . . . . . .    
36	C . . . . . . . . . .    
37	C . . . . . . . . . .    
38	T . . . . . . . . . .    
39	C . . . . . . . . . .    
40	T . . . . . . . . . .    
41	A . . . . . . . A . .    
42	G . . . . . . . G . .    
43	C . . . . . . . C . .    
44	A . . . . . . . A . .    
45	A . . . . . . . A . .    
46	T . . . . . . . T . .    
47	T . . . . . . . T . .    
48	G . . . . . . . G . .    
49	A . . . . . . . A . .    
50	C . . . . . . . C . .    
51	A . . . . . . . A . .    
52	C . . . . . . . C . .    
53	C . . . . . . . C . .    
54	T . . . . . . . T . .    
55	G . . . . . . . G . .    
56	T . . . . . . . T . .    
57	T . . . . . . . T . .    
58	C . . . . . . . C . .    
59	T . . . . . . . T . .    
60	C . . . . . . . C . .    
61	T . . . . . . . T . .    
62	G . . . . . . . G . .    
63	A . . . . . . . A . .    
64	T . . . . . . . T . .    
65	T . . . . . . . T . .    
66	G . . . . . . . G . .    
67	T . . . . . . . T . .    
68	G . . . . . . . G . .    
69	T . . . . . . . T . .    
70	T . . . . . . . T . .    
71	T . . . . . . T T . .    
72	T . . . . . . T T . .    
73	T . . . . . . T T . .    
74	A . . . . . . A A . .    
75	A . . . . . . A A . .    
76	C . . . . . . C C . .    
77	T . . . . . . T T . .    
78	T . . . . . . T T . .    
79	G . . . . . . G G . .    
80	T . . . . . . T T . .    
81	C . . . . . . C C . .    
82	C . . . . . . C C . .    
83	T . . . . . . T T . .    
84	T . . . . . . T T . .    
85	T . . . . . . T T . .    
86	T . . . . . . T T . .    
87	G . . . . . G G G . .    
88	T . . . . . T T T . .    
89	G . . . . . G G G . .    
90	T . . . . . T T T . .    
91	T . . . . . T T T . .    
92	C . . . . . C C C . .    
93	T . . . . . T T T . .    
94	G . . . . . G G G . .    
95	T . . . . . T T T . .    
96	A . . . . . A A A . .    
97	A . . . . . A A A . .    
98	A . . . . . A A A . .    
99	C . . . . . C C C . .    
100	A . . . . . A A A . .    
101	T . . . . . T T T . .    
102	A . . . . . A A A . .    
103	C . . . . . C C C . .    
104	C . . . . . C C C . .    
105	T T . . . . T T T . .    
106	A A . . . . A A A . .    
107	G G . . . . G G G . .    
108	A A . . . . A A A . .    
109	A A . . . . A A A . .    
110	A A . . . . A A A . .    
111	T T . . . . T T T . .    
112	G G . . . . G G G . .    
113	C C . . . . C C C . .    
114	A A . . . . A A A . .    
115	G G . . . . G G G . .    
116	T T . . . . T T T . .    
117	A A . . . . A A A . .    
118	A A . . . . A A A . .    
119	A A . . . . A A A . .    
120	C C . . . . C C C . .    
121	A A . . . . A A A . .    
122	T T . . . . T T T . .    
123	C C . . . . C C C . .    
124	A A . . . . A G A . .    
125	G G . . . . G G G . .    
126	C C . . . . C C C . .    
127	A A . . . . A A A . .    
128	C C . . . . C C C . .    
129	T T . . . T T T T . .    
130	G G . . . G G G G . .    
131	T T . . . T T T T . .    
132	A A . . . A A A A . .    
133	A A . . . A A A A . .    
134	A A . . . A A A A A .    
135	T T . . . T T T T T .    
136	G G . . . G G G G G .    
137	G G . . . G G G G G .    
138	T T . . . T T T T T .    
139	T T . . . T T T T T .    
140	T T . . . T T T T T .    
141	T T . . . T T T T T .    
142	T T . . . T T T T T .    
143	G G . . . G G G G G .    
144	G G . . . G G G G G .    
145	C C . . . C C C C C .    
146	A A . . . A A A A A .    
147	T T . . . T T T T T .    
148	T T . . . T T T T T .    
149	T T . . . T T T T T .    
150	T T . . . T T T T T .    
151	C C . . . C C C C C .    
152	T T . . . T T T T T .    
153	T T . . . T T T T T .    
154	T T . . . T T T T T .    
155	C C . . . C C C C C .    
156	C C . . . C C C C C .    
157	C C . . . C C C C C .    
158	C C . . . C C C C C .    
159	C C . . . C C C C C .    
160	C C . . . C C C C C .    
161	T T . . . T T T T T .    
162	T T . . . T T T T T .    
163	T T . . . T T T T T .    
164	C C . . . C C C C C .    
165	A A . . . A A A A A .    
166	A A . . . A A A A A .    
167	A A . . . A A A A A .    
168	A A . . . A A A A A .    
169	T T . . . T T T T T .    
170	T T . . . T T T T T .    
171	C C . . . C C C C C .    
172	T T . . . T T T T T .    
173	A A . . . A A A A A .    
174	C C . . . C C C C C .    
175	T T . . . T T T T T .    
176	A A . . . A A A A A .    
177	A A . . . A A A A A .    
178	T T . . . T T T T T .    
179	C C . . . C C C C C .    
180	T T . . . T T T T T .    
181	C C . . . C C C C C .    
182	A A . . . A A A A A .    
183	A A . . . A A A A A .    
184	G G . . . G G G G G .    
185	T T . . . T T T T T .    
186	G G . . . G G G G G .    
187	C C . . . C C C C C .    
188	G G . . . G G G G G .    
189	A A . . A A A A A A .    
190	A A . . A A A A A A .    
191	C C . . C C C C C C .    
192	T T . . T T T T T T .    
193	G G . . G G G G G G .    
194	T T . . T T T T T T .    
195	T T . . T T T T T T .    
196	C C . . C C C C C C .    
197	A A . . A A A A A A .    
198	T T . . T T T T T T .    
199	C C . . C C C C C C .    
200	T T . . T T T T T T .    
201	C C . . C C C C C C .    
202	T T . . T T T T T T .    
203	T T . T T T T T T T .    
204	C C . C C C C C C C .    
205	C C . C C C C C C C .    
206	C C . C C C C C C C .    
207	A A . A A A A A A A .    
208	A A . A A A A A A A .    
209	C C . C C C C C C C .    
210	A A . A A A A A A A .    
211	T T . T T T T T T T .    
212	A A . A A A A A A A .    
213	G G . G G G G G G G .    
214	A A . A A A A A A A .    
215	C C . C C C C C C C .    
216	A A . A A A A A A A .    
217	T T . T T T T T T T .    
218	A A . A A A A A A A .    
219	G G . G G G G G G G .    
220	T T . T T T T T T T .    
221	T T . T T T T T T T .    
222	A A . A A A A A A A .    
223	T T . T T T T T T T .    
224	T T . T T T T T T T .    
225	G G . G G G G G G G .    
226	A A . A A A A A A A .    
227	A A . A A A A A A A .    
228	A A . A A A A A A A .    
229	A A . A A A A C A A .    
230	C C . C C C C C C C .    
231	C C . C C C C C C C .    
232	A A . A A A A A A A .    
233	G G . G G G G G G G .    
234	A A . A A A A A A A .    
235	C C . C C C C C C C .    
236	A A . A A A A A A A .    
237	A A . A A A A A A A .    
238	A A . A A A A A A A .    
239	G G . G G G G G G G .    
240	C C . C C C C C C C .    
241	A A . A A A A A A A .    
242	T T . T T T T T T T .    
243	G G . G G G G G G G .    
244	A A . A A A A A A A .    
245	A A . A A A A A A A .    
246	A A . A A A A A A A .    
247	A A . A A A A A A A .    
248	G G . G G G G G G G .    
249	A A . A A A A A A A .    
250	A A . A A A A A A A .    
251	C C . C C C C C C C .    
252	A A . A A A A A A A .    
253	G G . G G G G G G G .    
254	A A . A A A A A A A .    
255	C C . C C C C C C C .    
256	A A . A A A A A A A .    
257	A A . A A A A A A A .    
258	T T . T T T T T T T .    
259	T T . T T T T T T T .    
260	C C . C C C C C C C .    
261	A A . A A A C C A A .    
262	C C . C C C C C C C .    
263	A A A A A A A A A A .    
264	A A A A A A A A A A .    
265	G G G G G G G G G G .    
266	G G G G G G G G G G .    
267	C C C C C C C C C C .    
268	T T T T T T T T T T .    
269	A A A A A A A A A A .    
270	A A A A A A A A A A .    
271	C C C C C C C C C C .    
272	C C C C C C C C C C .    
273	A A A A A A A A A A .    
274	C C C C C C C C C C .    
275	T T T T T T T T T T .    
276	G G G G G G G G G G .    
277	C C C C C C C C C C .    
278	A A A A A A A A A A .    
279	T T T T T T T T T T .    
280	T T T T T T T T T T .    
281	T T T T T T T T T T .    
282	G G G G G G G G G G .    
283	T T T T T T T T T T .    
284	G G G G G G G G G G .    
285	G G G G G G G G G G .    
286	C C C C C C C C C C .    
287	T T T T T T T T T T .    
288	A A A A A A A A A A .    
289	T T T T T T T T T T .    
290	A A A A A A A A A A .    
291	G G G G G G G G G G .    
292	G G G G G G G G G G .    
293	A A A A A A A A A A .    
294	G G G G G G G G G G .    
295	T T T T T T T T T T .    
296	T T T T T T T T T T .    
297	C C C C C C C C C C .    
298	T T T T T T T T T T .    
299	T T T T T T T T T T .    
300	G G G G G G G G G G .    
301	G G G G G G G G G G .    
302	A A A A A A A A A A .    
303	A A A A A A A A A A .    
304	A A A A A A A A A A .    
305	A A A A A A A A A A .    
306	G G G G G G G G G G .    
307	T T T T T T T T T T .    
308	A A A A A A A A A A .    
309	A A A A A A A A A A .    
310	T T T T T T T T T T .    
311	C C C C C C C C C C .    
312	A A A A A A A A A A .    
313	A A A A A A A A A A .    
314	T T T T T T T T T T .    
315	T T T T T T T T T T .    
316	G G G G G G G G G G .    
317	A A A A A A A A A A .    
318	A A A A A A A A A A .    
319	A A A A A A A A A A .    
320	G G G G G G G G G G .    
321	C C C C C C C C C C .    
322	T T T T T T T T T T .    
323	C C C C C C C C C C .    
324	A A A A A A A A A A .    
325	A A A A A A A A A A .    
326	T T T T T T T T T T .    
327	A A A A A A A A A A .    
328	C C C C C C C C C C .    
329	T T T T T T T T T T .    
330	A A A A A A A A A A .    
331	T T T T T T T T T T .    
332	T T T T T T T T T T .    
333	T T T T T T T T T T .    
334	A A A A A A A A A A .    
335	C C C C C C C C C C .    
336	A A A A A A A A A A .    
337	A A A A A A A A A A .    
338	A A A A A A A A A A .    
339	C C C C C C C C C C .    
340	T T T T T T T T T T .    
341	T T T T T T T T T T .    
342	A A A A A A A A A A .    
343	T T T T T T T T T T .    
344	G G G G G G G G G G .    
345	T T T T T T T T T T .    
346	T T T T T T T T T T .    
347	G G G G G G G G G G .    
348	T T T T T T T T T T .    
349	T T T T T T T T T T .    
350	G G G G G G G G G G .    
351	A A A A A A A A A A .    
352	A A A A A A A A A A .    
353	A A A A A A A A A A .    
354	A A A A A A A A A A .    
355	G G G G G G G G G G .    
356	G G G G G G G G G G G    
357	G G G G G G G G G G G    
358	A A A A A A A A A A A    
359	G G G G G G G G G G G    
360	A A A A A A A A A A A    
361	T T T T T T T T T T T    
362	C C C C C C C C C C C    
363	C C C C C C C C C C C    
364	G G G G G G G G G G G    
365	A A A A A A A A A A A    
366	T T T T T T T T T T T    
367	C C C C C C C C C C C    
368	A A A A A A A A A A A    
369	G G G G G G G G G G G    
370	T T T T T T T T T T T    
371	A A A A A A A A A A A    
372	T T T T T T T T T T T    
373	A A A A A A A A A A A    
374	C C C C C C C C C C C    
375	T T T T T T T T T T T    
376	G G G G G G G G G G G    
377	T T T T T T T T T T T    
378	G G G G G G G G G G G    
379	G G G G G G G G G G G    
380	T T T T T T T T T T T    
381	C C C C C C C C C C C    
382	C C C C C C C C C C C    
383	A A A A A A A A A A A    
384	T T T T T T T T T T T    
385	A A A A A A A A A A A    
386	T T T T T T T T T T T    
387	C C C C C C C C C C C    
388	A A A A A A A A A A A    
389	G G G G G G G G G G G    
390	T T T T T T T T T T T    
391	T T T T T T T T T T T    
392	C C C C C C C C C C C    
393	T T T T T T T T T T T    
394	G G G G G G G G G G G    
395	C C C C C C C C C C C    
396	C C C C C C C C C C C    
397	T T T T T T T T T T T    
398	T T T T T T T T T T T    
399	C C C C C C C C C C C    
400	A A A A A A A A A A A    
401	G G G G G G G G G G G    
402	T T T T T T T T T T T    
403	C C C C C C C C C C C    
404	A A A A A A A A A A A    
405	T T T T T T T T T T T    
406	C C C C C C C C C C C    
407	A A A A A A A A A A A    
408	A A A A A A A A A A A    
409	A A A A A A A A A A A    
410	C C C C C C C C C C C    
411	C C C C C C C C C C C    
412	A A A A A A A A A A A    
413	A A A A A A A A A A A    
414	C C C C C C C C C C C    
415	A A A A A A A A A A A    
416	T T T T T T T T T T T    
417	C C C C C C C C C C C    
418	A A A A A A A A A A A    
419	A A A A A A A A A A A    
420	T T T T T T T T T T T    
421	A A A A A A A A A A A    
422	G G G G G G G G G G G    
423	A A A A A A A A A A A    
424	A A A A A A A A A A A    
425	G G G G G G G G G G G    
426	G G G G G G G G G G G    
427	A A A A A A A A A A A    
428	A A A A A A A A A A A    
429	T T T T T T T T T T T    
430	A A A A A A A A A A A    
431	C C C C C C C C C C C    
432	T T T T T T T T T T T    
433	T T T T T T T T T T T    
434	T T T T T T T T T T T    
435	G G G G G G G G G G G    
436	G G G G G G G G G G G    
437	T T T T T T T T T T T    
438	C C C C C C C C C C C    
439	C C C C C C C C C C C    
440	T T T T T T T T T T T    
441	A A A A A A A A A A A    
442	T T T T T T T T T T T    
443	T T T T T T T T T T T    
444	G G G G G G G G G G G    
445	C C C C C C C C C C C    
446	A A A A A A A A A A A    
447	A A A A A A A A A A A    
448	G G G G G G G G G G G    
449	G G G G G G G G G G G    
450	A A A A A A A A A A A    
451	T T T T T T T T T T T    
452	G G G G G G G G G G G    
453	A A A A A A A A A A A    
454	T T T T T T T T T T T    
455	T T T T T T T T T T T    
456	C C C C C C C T C C C    
457	T T T T T T T T T T T    
458	G G G G G G G G G G G    
459	C C C C C C C C C C C    
460	T T T T T T T T T T T    
461	G G G G G G G G G G G    
462	C C C C C C C C C C C    
463	C C C C C C C C C C C    
464	A A A A A A A A A A A    
465	G G G G G G G G G G G    
466	T T T T T T T T T T T    
467	G G G G G G G G G G G    
468	A A A A A A A A A A A    
469	C C C C C C C C C C C    
470	A A A A A A A A A A A    
471	C C C C C C C C C C C    
472	C C C C C C C C C C C    
473	A A A A A A A A A A A    
474	C C C C C C C C C C C    
475	C C C C C C C C C C C    
476	T T T T T T T T T T T    
477	C C C C C C C C C C C    
478	C C C C C C C C C C C    
479	C C C C C C C C C C C    
480	T T T T T T T T T T T    
481	C C C C C C C C C C C    
482	A A A A A A A A A A A    
483	T T T T T T T T T T T    
484	G G G G G G G G G G G    
485	A A A A A A A A A A A    
486	C C C C C C C C C C C    
487	T T T T T T T T T T T    
488	C C C C C C C C C C C    
489	C C C C C C C C C C C    
490	A A A A A A A A A A A    
491	G G G G G G G G G G G    
492	T T T T T T T T T T T    
493	C C C C C C C C C C C    
494	A A A A A A A A A A A    
495	G G G G G G G G G G G    
496	T T T T T T T T T T T    
497	C C C C C C C C C C C    
498	A A A A A A A A A A A    
499	T T T T T T T T T T T    
500	T T T T T T T T T T T    
501	T T T T T T T T T T T    
502	A A A A A A A A A A A    
503	C C C C C C C C C C C    
504	T T T T T T T T T T T    
505	C C C C C C C C C C C    
506	C C C C C C C C C C C    
507	C C C C C C C C C C C    
508	A A A A A A A A A A A    
509	T T T T T T T T T T T    
510	G G G G G G G G G G G    
511	A A A A A A A A A A A    
512	T T T T T T T T T T T    
513	G G G G G G G G G G G    
514	A A A A A A A A A A A    
515	G G G G G G G G G G G    
516	C C C C C C C C C C C    
517	G G G G G G G G G G G    
518	A A A A A A A A A A A    
519	A A A A A A A A A A A    
520	T T T T T T T T T T T    
521	G G G G G G G G G G G    
522	C C C C C C C C C C C    
523	T T T T T T T T T T T    
524	C C C C C C C C C C C    
525	T T T T T T T T T T T    
526	C C C C C C C C C C C    
527	C C C C C C C C C C C    
528	T T T T T T T T T T T    
529	T T T T T T T T T T T    
530	T T T T T T T T T T T    
531	A A A A A A A A A A A    
532	A A A A A A A A A A A    
533	A A A A A A A A A A A    
534	G G G G G G G G G G G    
535	T T T T T T T T T T T    
536	C C C C C C C C C C C    
537	C C C C C C C C C C C    
538	A A A A A A A A A A A    
539	C C C C C C C C C C C    
540	T T T T T T T T T T T    
541	T T T T T T T T T T T    
542	C C C C C C C C C C C    
543	A A A A A A A A A A A    
544	T T T T T T T T T T T    
545	C C C C C C C C C C C    
546	G G G G G G G G G G G    
547	T T T T T T T T T T T    
548	A A A A A A A A A A A    
549	A A A A A A A A A A A    
550	G G G G G G G G G G G    
551	A A A A A A A A A A A    
552	A A A A A A A A A A A    
553	C C C C C C C C C C C    
554	A A A A A A A A A A A    
555	A A A A A A A A A A A    
556	C C C C C C C C C C C    
557	A A A A A A A A A A A    
558	T T T T T T T T T T T    
559	G G G G G G G G G G G    
560	T T T T T T T T T T T    
561	A A A A A A A A A A A    
562	G G G G G G G G G G G    
563	T T T T T T T T T T T    
564	G G G G G G G G G G G    
565	G G G G G G G G G G G    
566	T T T T T T T T T T T    
567	T T T T T T T T T T T    
568	T T T T T T T T T T T    
569	C C C C C C C C C C C    
570	A A A A A A A A A A A    
571	T T T T T T T T T T T    
572	T T T T T T T T T T T    
573	T T T T T T T T T T T    
574	- A A A A A A A - - -  4	 indel  
575	- T T T T T T T - - -  4	 indel  
576	G G G G G G G G G G G    
577	T T T T T T T T T T T    
578	T T T T T T T T T T T    
579	T T T T T T T T T T T    
580	T T T T T T T T T T T    
581	G G G G G G G G G G G    
582	T T T T T T T T T T T    
583	T T T T T T T T T T T    
584	T T T T T T T T T T T    
585	G G G G G G G G G G G    
586	T T T T T T T T T T T    
587	A A A A A A A A A A A    
588	T T T T T T T T T T T    
589	T T T T T T T T T T T    
590	C C C C C C C C C C C    
591	C C C C C C C C C C C    
592	T T T T T T T T T T T    
593	G G G G G G G G G G G    
594	G G G G G G G G G G G    
595	A A A A A A A A A A A    
596	A A A A A A A A A A A    
597	T T T T T T T T T T T    
598	T T T T T T T T T T T    
599	T T T T T T T T T T T    
600	C C C C C C C C C C C    
601	A A A A A A A A A A A    
602	A A A A A A A A A A A    
603	T T T T T T T T T T T    
604	G G G G G G G G G G G    
605	C C C C C C C C C C C    
606	G G G G G G G G G G G    
607	G G G G G G G G G G G    
608	T T T T T T T T T T T    
609	T T T T T T T T T T T    
610	G G G G G G G G G G G    
611	G G G G G G G G G G G    
612	T T T T T T T T T T T    
613	T T T T T T T T T T T    
614	G G G G G G G G G G G    
615	G G G G G G G G G G G    
616	A A A A A A A A A A A    
617	T T T T T T T T T T T    
618	A A A A A A A A A A A    
619	A A A A A A A A A A A    
620	T T T T T T T T T T T    
621	G G G G G G G G G G G    
622	G G G G G G G G G G G    
623	G G G G G G G G G G G    
624	A A A A A A A A A A A    
625	C C C C C C C C C C C    
626	A A A A A A A A A A A    
627	T T T T T T T T T T T    
628	T T T T T T T T T T T    
629	T T T T T T T T T T T    
630	A A A A A A A A A A A    
631	A A A A A A A A A A A    
632	C C C C C C C C C C C    
633	T T T T T T T T T T T    
634	G G G G G G G G G G G    
635	T T T T T T T T T T T    
636	A A A A A A A A A A A    
637	A A A A A A A A A A A    
638	A A A A A A A A A A A    
639	G G G G G G G G G G G    
640	A A A A A A A A A A A    
641	A A A A A A A A A A A    
642	A A A A A A A A A A A    
643	G G G G G G G G G G G    
644	A A A A A A A A A A A    
645	G G G G G G G G G G G    
646	G G G G G G G G G G G    
647	G G G G G G G G G G G    
648	A A A A A A A A A A A    
649	A A A A A A A A A A A    
650	T T T T T T T T T T T    
651	C C C C C C C C C C C    
652	A A A A A A A A A A A    
653	T T T T T T T T T T T    
654	A A A A A A A A A A A    
655	A A A A A A A A A A A    
656	C C C C C C C C C C C    
657	A A A A A A A A A A A    
658	T T T T T T T T T T T    
659	T T T T T T T T T T T    
660	C C C C C C C C C C C    
661	C C C C C C C C C C C    
662	A A A A A A A A A A A    
663	G G G G G G G G G G G    
664	T T T T T T T T T T T    
665	A A A A A A A A A A A    
666	T T T T T T T T T T T    
667	A A A A A A A A A A A    
668	A A A A A A A A A A A    
669	C C C C C C C C C C C    
670	A A A A A A A A A A A    
671	T T T T T T T T T T T    
672	T T T T T T T T T T T    
673	T T T T T T T T T T T    
674	G G G G G G G G G G G    
675	A A A A A A A A A A A    
676	T T T T T T T T T T T    
677	G G G G G G G G G G G    
678	A A A A A A A A A A A    
679	A A A A A A A A A A A    
680	A A A A A A A A A A A    
681	A A A A A A A A A A A    
682	A A A A A A A A A A A    
683	G G G G G G G G G G G    
684	A A A A A A A A A A A    
685	T T T T T T T T T T T    
686	C C C C C C C C C A C    
687	. . . . . . . . . T .    
688	. . . . . . . . . T .    
689	. . . . . . . . . T .    
690	. . . . . . . . . A .    
691	. . . . . . . . . A .    
692	. . . . . . . . . T .    
693	. . . . . . . . . T .    
694	. . . . . . . . . G .    
695	. . . . . . . . . A .    
696	. . . . . . . . . T .    
697	. . . . . . . . . T .    
698	. . . . . . . . . A .    
699	. . . . . . . . . A .    
700	. . . . . . . . . A .    
701	. . . . . . . . . G .    
702	. . . . . . . . . T .    
703	. . . . . . . . . G .    
704	. . . . . . . . . T .    
705	. . . . . . . . . A .    
706	. . . . . . . . . T .    
707	. . . . . . . . . T .    
708	. . . . . . . . . A .    
709	. . . . . . . . . T .    
710	. . . . . . . . . T .    
711	. . . . . . . . . C .    
712	. . . . . . . . . G .
```
